# Rosmarinic Acid Reduces Microglia Senescence: A Novel Therapeutic Approach for the Management of Neuropathic Pain Symptoms

**DOI:** 10.3390/biomedicines10071468

**Published:** 2022-06-21

**Authors:** Vittoria Borgonetti, Nicoletta Galeotti

**Affiliations:** Department of Neuroscience, Psychology, Drug Research and Child Health (NEUROFARBA), Section of Pharmacology, University of Florence, 50139 Florence, Italy; vittoria.borgonetti@unifi.it

**Keywords:** microglia, senescence, neuropathic pain, rosmarinic acid, polyphenols

## Abstract

The worldwide incidence of neuropathic pain is around 7–8% and is associated with significant and disabling comorbidities (sleep disturbances, depression, anxiety). It is now known that cellular ageing of microglia contributes to neurodegenerative diseases, mood disorders, and, even if with less evidence, chronic pain. The aim of this work was to investigate in vitro and in vivo the senolytic activity of rosmarinic acid (RA) to be exploited for the management of NP symptoms. BV2 cells were stimulated with LPS 500 ng/mL for 24 h. Treatment with RA 1 µM improved cell viability and reduced IL-1ß release leading to an attenuation of neuroinflammation. We then moved on to test the efficacy of RA in reducing microglial senescence. In our model, BV2 cells were stimulated with LPS 500 ng/mL every 72 h for 4 h/day, over a period of 10 days. RA 1 µM reduced the expression of the β-galactosidase enzyme, reduced the release of senescence-associated secretory phenotype (SASP) factors, increased cell viability, and reduced the presence of nuclear foci of senescence (SAHF), well-known cellular senescence markers. In the Spared Nerve Injury (SNI) model, 28 days from surgery, repeated oral administration of RA 5 mg/kg reduced hyperalgesia and NP-associated symptoms, such as anxiety and depression. A reduction of senescence markers was detected on both hippocampal and spinal samples of SNI-treated mice. This study represents a starting point for investigating the role of microglial senescence as a possible pharmacological target in controlling symptoms related to the more advanced stages of peripheral neuropathy.

## 1. Introduction

The worldwide incidence of neuropathic pain (NP) is around 7–8% and is associated with significant and disabling comorbidities, such as sleep disturbances, depression, and anxiety, which further affect patients’ quality of life [1]. Controversies over the most appropriate therapeutic approach still exist, as neuropathy is a multifactorial and subjective condition. In fact, there are numerous side effects associated with drug treatments, including sedation related to the use of tricyclic antidepressants (TCAs) or gabapentin/pregabalin, and to the anticholinergic activity of TCAs [2]. The use of medicinal plant preparations for the treatment of neuropathies has been gaining momentum in recent years, especially considering their increased patient compliance. One of the most extensively researched medicinal plants is *Melissa officinalis* L., which has been used for centuries for its sedative effects; preparations based on it are known to have anxiolytic and antidepressant properties, which are mainly due to the presence of rosmarinic acid (RA) [3,4,5,6]. RA is known for its antioxidant, antibacterial against both gram-positive and gram-negative bacteria, antiviral, anti-inflammatory, analgesic, neuroprotective, cardioprotective, and many other activities [7]. The anti-inflammatory activity of RA may be exploited for the management of NP, since it represents a chronic neuroinflammatory disease, consisting of a strong microglial activation. NP begins with an inflammatory process, which if unresolved can lead to alteration of the normal activity of the central nervous system environment. In this condition, the microglia may not withstand this continuous stimulation and lose their function, until they become senescent [8]. It was recently shown that senescent microglia are characterized by cellular hyperactivation leading to morphological change, reduced phagocytic activity, and massive release of pro-inflammatory cytokines [9,10]. It is now known that cellular ageing of microglia contributes to neurodegenerative diseases, mood disorders, and, even if with less evidence, chronic pain [11]. Under normal conditions, microglia cells are activated in response to a stimulus or insult to the body, developing a physiological inflammatory response to maintain homeostasis [12]. However, as in many pathological contexts, the inflammatory process tends to become chronic due to excessive microglial activation, with accelerated ageing and loss of function [13]. Finding a therapy that can control pain and comorbidities is certainly an important key point in the therapeutic approach of neuropathies. The aim of this work is to test the effectiveness of RA in reducing the symptomatology associated with peripheral neuropathy in the spared nerve injury (SNI) model by reducing microglia senescence.

## 2. Materials and Methods

### 2.1. BV2

BV-2 (murine microglial cells, C57BL/6 Tema Ricerca, Genova, Italy) cells were used for this study. Cells were kept in culture in a 75 cm^2^ flask (Sarstedt, Verona, Italy) in medium containing RPMI with 10% heat-inactivated fetal bovine serum (56 °C, 30 min) (FBS, Gibco^®^, Milan, Italy), 1% glutamine, and 1% penicillin-streptomycin solution (Merck, Milan, Italy). Cells were cultured at 37 °C and 5% CO_2_ with daily change of culture medium. For the neuroinflammatory and senescent model, we used bacterial lipopolysaccharide from Gram- (LPS, *Salmonella enteridis*, Merck, Darmstadt, Germany) solubilized in RPMI to obtain a stock of 500 µg/mL and diluted in the medium of BV2 cells to obtain a final concentration of 500 ng/mL. Briefly, for the neuroinflammation model, BV2 were treated continuously for 24 h with LPS 500 ng/mL, in minimal medium (RPMI with 3% FBS). For the senescence model, BV2 cells were treated 4 times, for 4 h/day for a total of 10 days with LPS 500 ng/mL in minimal medium (RPMI with 3% FBS). RA (Merck, Darmstadt, Germany) was solubilized directly in RPMI cell culture medium at a concentration of 1 mg/mL, filtered (Filter syringe 0.2 μm, 30 mm, Biosigma, Venice, Italy), and then diluted in the medium to obtain final concentrations of 0.01, 0.1, 1, and 10 µM. In the neuroinflammatory model, RA was added 6 h after the starting of the LPS stimulation. In the microglial senescence model, a 4 h pre-treatment of the cells with RA was performed on the last 3 days of the total time interval, followed by stimulation with LPS [14].

### 2.2. Sulforhodamine B (SRB) Assay

The viability of the cells was assessed by the SRB test. Briefly, cells were seeded in 96-well plates (2 × 10^4^ cells per well). After treatment, cells were fixed in 50% trichloroacetic acid (TCA, Merck, Darmstadt, Germany) in RPMI at 4 °C for 1 h. The next day, the wells were treated with a 4 mg/mL solution of SRB in 1% acetic acid in double distilled H_2_O for 30 min at rt. Then, 4/5 washes with acetic acid were performed to remove excess dye. Finally, Tris-HCl (pH = 10) was used for cell lysis and absorbance at 570 nm was recorded using a multiplate reader (Biorad, Milan, Italy). The treatments were carried out in three independent experiments (*n* = 3), and cell viability was calculated by normalizing the values to the mean of the control [15].

### 2.3. Senescence-Associated Heterochromatic Foci Analysis (SAHF)

BV2 cells were initially seeded in 24-well plates (10 × 10^4^ cells per well) containing previously sterilized (brand) slides at the bottom of the wells. After appropriate treatments, cells were fixed with 4% PFA for 30 min at 4 °C. After 3 washes with PBS, the slides were treated with a solution of 1 μg/mL DAPI in mounting medium (90% glycerol + PBS), allowed to dry in the dark at rt overnight, and then stored at 20 °C. After approximately one week, the slides were observed with an OLYMPUS BX63F fluorescence microscope connected to a PC with an image acquisition card. The treatments were carried out in three independent experiments (*n* = 3), and the DAPI intensity was calculated by normalizing the values to the mean of the control [16].

### 2.4. Animals

CD1 male mice (8 weeks of age, 20 g, Envigo, Varese, Italy) were housed in the vivarium of Ce.S.A.L. (Centro Stabulazione Animali da Laboratorio, University of Florence, Florence, Italy) and used seven days after their arrival. Mice were housed in standard cages, maintained at 23 ± 1 °C with a 12 h light/dark cycle, light on at 7 am, and fed with standard laboratory diet and tap water ad libitum. All tests were conducted during the light phase. The experimental protocol was approved by the ethical committee for animal care and research of the institute (University of Florence), under license of the Italian Ministry of Health (410/2017-PR). Mice were handled in accordance with relevant European Union (Council Directive 2010/63/EU of 22 September 2010 on the protection of animals used for scientific purposes) and international regulations (Guide for the Care and Use of Laboratory Animals, US National Research Council, 2011). All studies involving animals are reported in accordance with the ARRIVE 2.0 guidelines for experiments involving animals. The experimental protocol was designed to minimize the number of animals used and their suffering. G power software was used to perform a power analysis to choose the number of animals per experiment [17,18].

### 2.5. SNI Procedure Experimental Schedule

The SNI is a model of peripheral mono-neuropathy, which was performed as previously described. Briefly, animals were anaesthetized with 4% isoflurane in O_2_/N_2_O (30: 70 *v*/*v*). The right paw, conventionally referred to as ‘ipsi’, was operated, while the left ‘contra’ was left intact. In the trifurcation of the sciatic nerve, the peroneal and tibial were tied together with 5.0 silk thread (Ethicon; Johnson & Johnson Intl, Brussels, Belgium) and cut, while the sural remained intact. Tests were conducted 7 days after the operation to observe operation-induced thermal hyperalgesia. Sex differences in pain response were described in the SNI model with microgliosis and hypersensitivity to pain being mainly found in male mice. RA (1, 5, and 10 mg/kg, p.o.) was solubilized in saline and orally administered (*n* = 8) via gavage daily, once a day, starting from day 21 to 28 after surgery. The control un-operated (Sham) and operated (SNI) groups were treated with the vehicle (*n* = 8) [19,20].

### 2.6. Acute Pain and Thermal Hyperalgesia Measured with the Hot Plate Test

To induce an acute stimulus in the model of acute pain and to assess thermal hyperalgesia in the SNI model, the hot plate test was used. Briefly, the animal’s response time to a thermal stimulus (52 °C) applied to a 24 cm-diameter electrically operated device was measured. Mice were placed on the hot plate, surrounded by a transparent acrylic cage. A response was considered positive when the animals licked themselves, shacked their heads or jumped. In SNI mice, this test was performed in the baseline (BL) condition and at day 7-21-28 after surgery [21].

### 2.7. Rotarod Test

The possible onset of motor side effects induced by treatment was evaluated with rotarod test, as previously described [22]. Animals were habituated before starting the test. The rotarod is an instrument consisting of a rotating rod with a diameter of approximately 5 cm. This rod is placed at a height of about 15 cm from the base of the instrument. The speed of the rod was 16 rpm and the time in which the number of falls of the animal was calculated was 30 s. This test was performed in the baseline (BL) condition and at day 21–28 after surgery [23].

### 2.8. Evaluation of Anxiolytic-like Effect

#### 2.8.1. Open Field (OF) Test

The open field test was used to assess anxiety levels in the animal. A rectangular box (78 cm × 60 cm × 39 cm) was used, in which an inner perimeter approximately 3 cm from the walls was defined. The animals were placed in the center of the box and habituated for 5 min. After, the time the animal remained in the inner portion during the 5-min test was measured. The longer the animal remained in the center of the box, the less anxious it was. This test was performed in the baseline condition (BL) and on day 21–28 after surgery [23].

#### 2.8.2. Light Dark Box (LDB) Test

The LDB test was performed as previously reported [7]. Briefly, each mouse was left free to move for 5 min in a box with two different compartments, the dark and the light (60-W bulb lamp, white) chamber, separated by a small door (10 cm × 3.2 cm). The time spent in the light compartment and the number of transitions was used as a sign of the anxiety state of each animal. This test was performed on day 21–28 after surgery [23].

### 2.9. Evaluation of Antidepressant-like Activity

#### 2.9.1. Sucrose Splash Test (SST)

The aim of the test was to assess the presence of a depressive-like behavior in the mouse. A 10% sucrose solution in H_2_O was prepared and a small amount was placed on the animal’s back. The mouse was placed inside a box, and the time it spent cleaning was measured over the total test duration of 5 min. The aim was to obtain information on the state of depression of the mouse; the more marked this was, the more the animal tended to clean itself with difficulty. This test was performed in the baseline condition (BL) and at day 21–28 after surgery [24].

#### 2.9.2. Tail Suspension Test (TST)

The TST was performed as described by Borgonetti and co-authors [9]. Mice were suspended from a pole attached 50 cm overhead the base by means of adhesive tape placed in the middle of the tail. The time during which the mice persisted immobile was measured with a stopwatch during a 6 min test period. Mice were considered immobile when they drooped impassively and entirely unmoving, except for movements caused by breathing. Immobility was considered as a depression-like behavior (behavioral despair) and was measured in the last 4 min, when behavioral despair is established, and an antidepressant activity of a drug is more easily identified. This test was performed at day 21–28 after surgery [24].

### 2.10. Cells and Tissues Protein Extraction

Proteins from adherent cells were extracted by RIPA buffer (50 mM Tris-HCl pH 7.4, 150 mM NaCl 1% sodium deoxycolate, 1% Tryton X-100, 2 mM PMSF) (Sigma-Aldrich, Milan, Italy) and the insoluble pellet was separated by centrifugation (12,000× *g* for 30 min, 4 °C).

To detect the release of IL-1ß, we used the media of BV2 cells. From the cell medium, 50% TCA was added and left to incubate at 4 °C for 10 min. This was followed by centrifugation at 14,000 rpm for 5 min, after which the supernatant was removed. The pellet (precipitated proteins) was then resuspended in cold acetone and the microtubes placed in an oven to promote evaporation of the acetone and drying of the pellet (60 °C approx. for 20 min). Finally, the pellet was resuspended in 4X Loading Buffer (Merck, Darmstadt, Germany) and the finished samples were stored at −20 °C.

To examine protein expression in animals, tissues were removed after 28 days from surgery. Samples were homogenized in a lysis buffer containing 25 mM Tris-HCl pH (7.5), 25 mM NaCl, 5 mM EGTA, 2.5 mM EDTA, 2 mM NaPP, 4 mM PNFF, 1 mM di Na3VO4, 1 mM PMSF, 20 μg/mL leupeptin, 50 μg/mL aprotinin, 0.1% SDS (Merck, Darmstadt, Germany). The homogenate was centrifuged at 12,000× *g* for 30 min at 4 °C and the pellet was discarded. The total protein concentration in the supernatant was measured using Bradford colorimetric method (Merck, Darmstadt, Germany) [14,25].

### 2.11. Western Blotting

Protein samples (30 µg of protein/sample) were separated by 10% SDS-polyacrylamide gel electrophoresis (SDS-PAGE). Proteins were then blotted onto nitrocellulose membranes (90 min at 110 V) using standard procedures. Membranes were blocked in PBST (PBS with 0.1% Tween) containing 5% non-fat dry milk for 90 min and incubated overnight at 4 °C with primary antibodies: anti-ß-galactosidase (Santa Cruz Biotechnology, Dallas, TX, USA, Cat# sc-377257), anti-IκBα (Santa Cruz Biotechnology, Dallas, TX, USA, Cat# sc-1643, RRID:AB_627772), and anti-IL-1ß (Santa Cruz Biotechnology, Dallas, TX, USA, Cat# sc-52012, RRID:AB_629741). The day after, blots were rinsed three times with PBST and incubated for 2 h at rt with HRP-conjugated secondary antibodies and then detected by chemiluminescence detection system (Life Technologies Italia, Monza, Italy). Signal intensity (pixels/mm^2^) was quantified using ImageJ (NIH). The signal intensity was normalized to that of GAPDH (1:5000 Santa Cruz Biotechnology, Dallas, TX, USA) [25]. The treatments were carried out in three independent experiments (*n* = 4), and protein expression was calculated by normalizing the values to the mean of the control [20].

### 2.12. Statistical Analysis

For in vitro experiments and behavioral experiments, statistical analysis was obtained with one-way or two-way ANOVA, followed by Tukey or Bonferroni post hoc test. For each test, a value of *p* < 0.05 was considered significant. Data are expressed as the mean ± SEM. The software GraphPad Prism (version 5.0, San Diego, CA, USA) was used in all statistical analysis.

## 3. Results

### 3.1. RA Reduced LPS-Induced Toxicity and IL-1β Release in BV2 Microglia Cells

**Figure 1:**
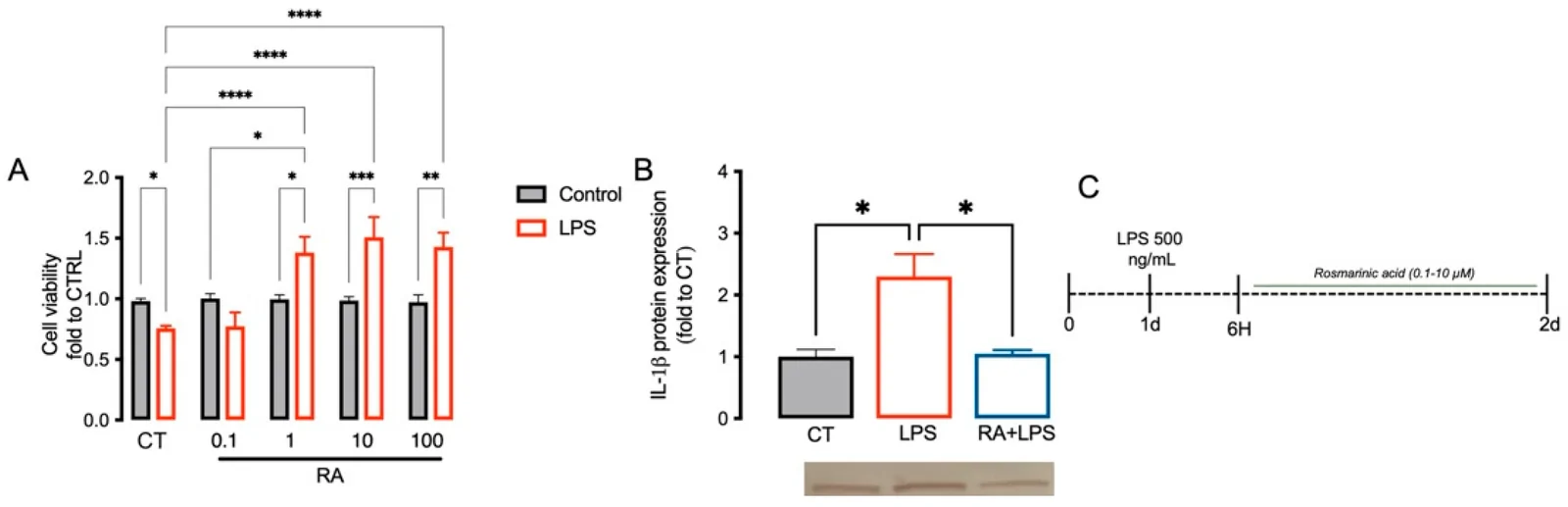
Effect of RA on LPS-stimulated BV2 cells. (**A**) Evaluation of the effect of rosmarinic acid (RA, 0.1–10 µM) on counteracting LPS-induced toxicity on BV2. One-way ANOVA **** *p* < 0.0001 *** *p* < 0.001 ** *p* < 0.01 * *p* < 0.05 (*n* = 3). (**B**) Effect of RA 1 µM in reducing IL-1β release in LPS-stimulated BV2. One-way ANOVA * *p* < 0.05 (*n* = 3). (**C**) Schematic representation of the experimental protocol adopted in the neuroinflammation model.

RA is a polyphenol with anti-inflammatory and antioxidant activity [7]. In this work, we tested its ability to reduce the toxicity produced by stimulation with LPS 500 ng/mL in BV2 cells, to reproduce an in vitro model of neuroinflammation (Figure 1C). LPS 500 ng/mL reduced cell viability after 24 h of stimulation. Treatment with RA 0.01 µM was not able to counteract the toxic effect produced by LPS. The effect of RA was observed at a concentration of 0.1 µM and peaked at a concentration of 1 µM. No further increase was detected at higher doses (Figure 1A). LPS 500 ng/mL induced the phenotype shift of microglial cells, leading them to assume a pro-inflammatory phenotype. Indeed, after 24 h, LPS-stimulated BV2 showed an increase of IL-1β release in the media, compared to non-stimulated control (CT) cells (cells without any treatment). Treatment with RA 1 µM reduced microglial activation, as showed by a reduction of IL-1β release (Figure 1B), thus leading to an attenuation in the inflammatory process.

### 3.2. RA Reduced the Senescence Process in BV2 Cells Intermittently Stimulated with LPS

**Figure 2:**
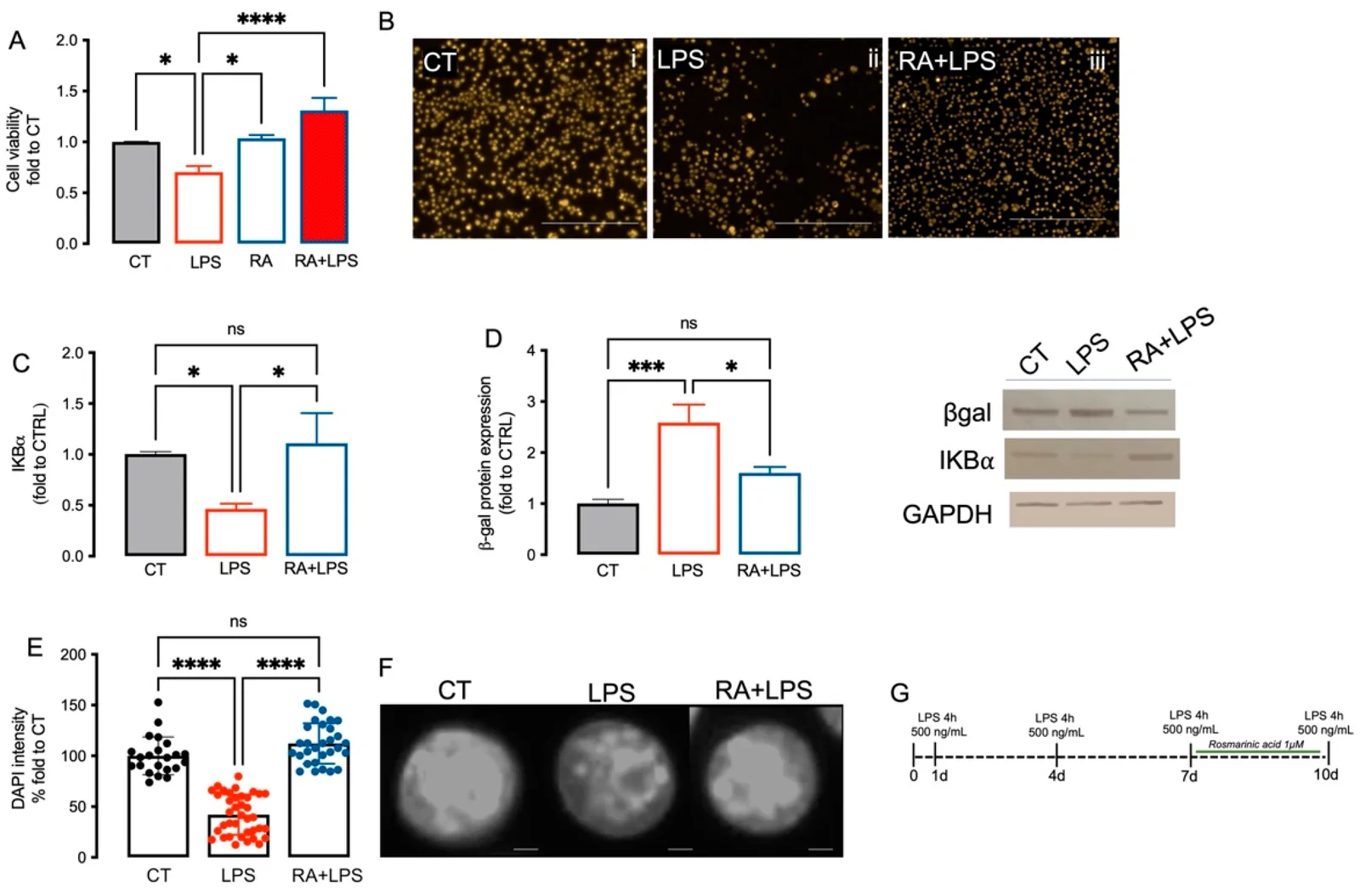
Evaluation of the effect of rosmarinic acid (RA) on microglial senescence. (**A**) Effect of RA 1 µM on cell viability. (**B**) Representative images of cell viability of untreated BV2 cells ((i); CT), senescent microglia ((ii), LPS) and pretreated-senescent BV2 cells ((iii), RA + LPS). Scale bar 200 µm). (**C**) IkBα protein expression. (**D**) ß-galactosidase (ß-gal) protein expression. (**E**) formation of SAHF in BV2 senescent cells at 10 days. Dots are the number of nuclei counted. One-way ANOVA **** *p* < 0.0001 *** *p* < 0.001, * *p* < 0.05 (*n* = 3). (**F**) Representative images of DAPI intensity. Scale bar 2 µm. (**G**) Schematic representation of the experimental protocol adopted in the neuroinflammation model. Representative blots are reported (*n* = 3). ns = not significant.

Intermittent repeated stimulation with an inflammatory agent such as LPS has been reported to induce senescence in microglial cells [14]. The senescence model was optimized by stimulating BV-2 murine microglial cells with LPS 500 ng/mL for 4 h/day for a total of 10 days (Figure 2G). The parameters we considered were cell viability and morphology, expression of inflammatory factors related to the senescence-associated secretory phenotype (SASP), expression of β-galactosidase (β-gal), and development of nuclear senescence foci. These parameters represent selective markers of microglial senescence [10]. Cells were pretreated with RA at the optimal concentration of 1 µM for 4 h on three consecutive days prior to day 10 of stimulation (i.e., on day 8, 9, and 10). Cell viability was considered in the optimization of the model, as senescent cells undergo cell death faster and have less capacity to easily reproduce. The SRB test showed that pre-treatment with RA can counteract the reduction in cell viability produced by LPS in BV2 cells, as not only was the level of cell viability restored to that of the controls, but there is even an increase in cell viability, indicating a potential protective effect (Figure 2A). A representative image of one well of CT ((i); unstimulated BV2), LPS ((ii); LPS-stimulated BV2), and RA + LPS ((iii); LPS-stimulated BV2 and treated with RA) is shown in Figure 2B. It is evident that the number of cells in the well treated with LPS was lower than in CT, while treatment with RA can increase the number of cells, consistently with what was found in the cell viability test. Senescent cells can interact with their environment by releasing inflammatory factors. These characterize the senescence-associated secretory phenotype or SASP [26].

NF-κB is part of these bioactive elements. LPS 500 ng/mL leads to a gradual decrease of the IκBα inhibitor at 10 days of stimulation (Figure 2C). β-gal represents one of the main markers associated with cellular ageing [27]. In fact, LPS 500 ng/mL increased β-gal expression in cells. The RA pretreatment resulted in a significant reduction in the levels of β-gal compared to the LPS-treated group (Figure 2D). Finally, Figure 2E,F show the development of senescence foci at the nuclear level. Fluorescence microscopy highlighted that the nucleus of cells not treated with inflammatory stimuli was homogeneous in shape and density (Figure 2E,F). On the contrary, the nucleus of LPS-stimulated cells was more fragmented and showed gaps corresponding to foci of senescence, a sign of an ongoing cellular ageing process (Figure 2E,F; [28]). Treating cells with LPS 500 ng/mL resulted in a progressive increase in the percentage of foci in the nucleus. Pretreatment with RA markedly reduced the presence of foci, making the core more uniform and similar to the control group (Figure 2E,F).

### 3.3. Analgesic Effect of RA in a Mice Model of Acute Pain

**Figure 3:**
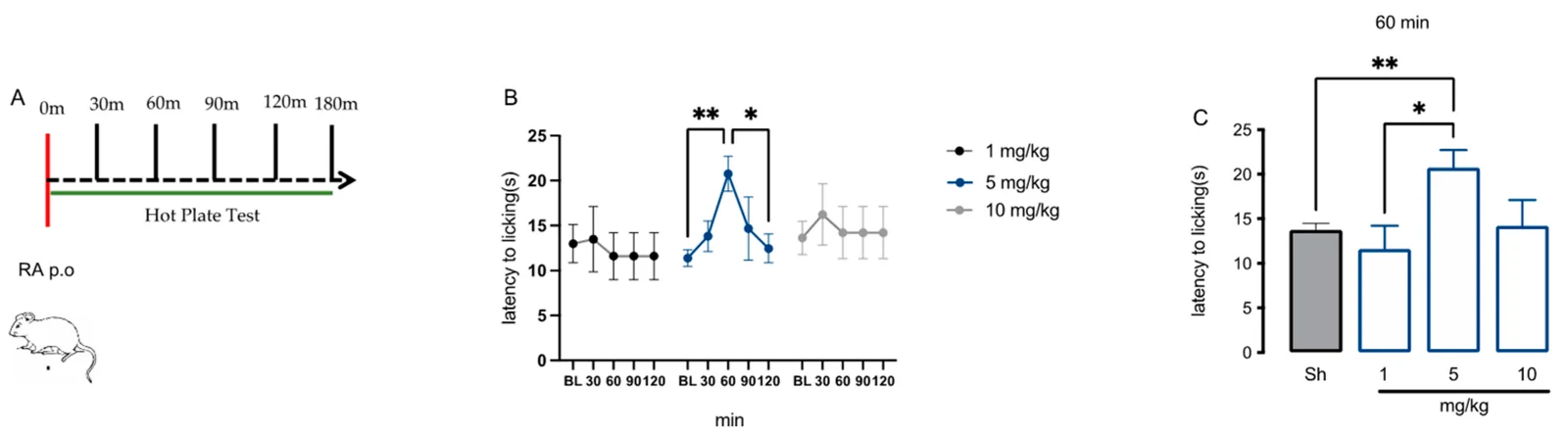
Effect of RA in acute pain. (**A**) Schematic representation of the protocol used in naive mice (Sham, Sh) in the acute pain model (*n* = 8 for each experimental group). (**B**) Dose-response curve of orally administered (p.o.) rosmarinic acid (RA) at 30, 60, 90, and 120 min, obtained by hot plate test. Two-way ANOVA ** *p* < 0.01, * *p* < 0.05 (*n* = 8). (**C**) Comparison of the effect obtained by RA at the dose of 1, 5, and 10 mg/kg at the 60-min peak activity. One-way ANOVA ** *p* < 0.01, * *p* < 0.05 (*n* = 8).

Once the effect of RA on neuroinflammation and microglial senescence had been assessed in vitro, we moved on to in vivo analysis. To evaluate the analgesic effect of RA in acute pain, the hot plate test was used (Figure 3A). A time course of analgesic activity was constructed for each dose by performing the test 30, 60, 90, and 120 min after oral administration. RA did not produce any effect at the doses of 1 mg/kg and 10 mg/kg (Figure 3B). At the dose of 5 mg/kg, RA increased the pain threshold 60 min after oral administration, disappearing at 90 and 120 min after administration. Analgesic activity peaked 60 min after administration and then faded at 90 and 120 min after administration (Figure 3B). Comparing the effect of a dose of 1 mg/kg, 5 mg/kg, and 10 mg/kg at 60 min after oral administration, RA showed a bell-shaped dose-response curve, typical of substances of natural origin (Figure 3C). We therefore used the RA effective dose obtained from this test in the other tests in the chronic model.

### 3.4. Effect of RA on Hyperalgesia and Associated Comorbidities in the SNI Model

**Figure 4:**
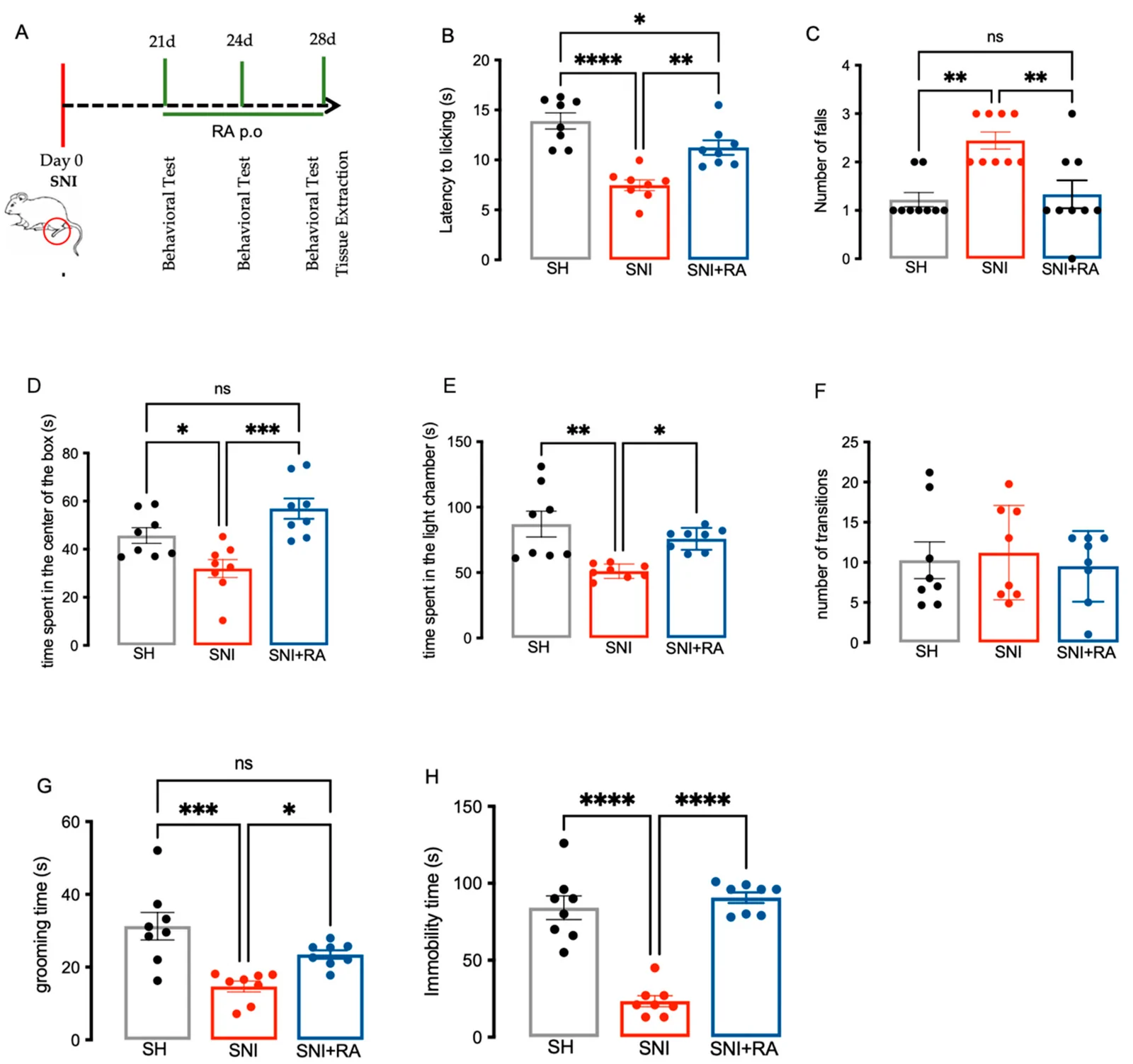
Effect of RA on SNI-induced behavioral phenotype. (**A**) Representation of the protocol used in the Spared Nerve Injury (SNI) model, in which the experimental group used are: unoperated mice (SH), operated mice (SNI), operated mice treated orally (p.o.) with rosmarinic acid (RA) 5 mg/kg (*n* = 8 for each experimental group). Evaluation of thermal hyperalgesia with hot plate test (**B**), locomotor function with rotarod (**C**), anxiety-like symptoms with open field test (**D**), and light-dark box (**E**): time spent in the light chamber, (**F**): number of transitions), depressive-like symptoms with sucrose splash test (**G**), and tail suspension test (**H**), final 4 min) after three weeks from surgery in SH, SNI, and SNI + RA. Dots are number of animals. ns = not significant. One-way ANOVA **** *p* < 0.0001, *** *p* < 0.001, ** *p* < 0.01, * *p* < 0.05.

RA was evaluated in a model of peripheral NP (SNI) (Figure 4A). RA was administered at a dose of 5 mg/kg, which was demonstrated to be the most effective dose from the dose–effect curve in acute pain, and the tests were performed after 60 min, identified as the time of peak activity (Figure 4A). Between 21 and 28 days after the operation, the animals were subjected to several behavioral tests monitoring: hyperalgesia, locomotor activity, anxiety-like state, and depression-like state. SNI animals developed severe hyperalgesia produced by sciatic nerve ligation. Daily oral administration of RA 5 mg/kg reduced the state of hyperalgesia produced by the model with values comparable to those of the non-operated animals (Sham). The control SNI animals were treated only with the vehicle in which the RA was dispersed (Figure 4B). Once the activity was demonstrated in pain, we moved towards the evaluation of the main comorbidities associated with neuropathic condition. The SNI animals also developed motor impairment, measured by the rotarod test, as the SNI animals fell more times in 30 s than the Sham animals. Animals treated with RA can reduce the number of falls, probably because they feel less pain (Figure 4C). To evaluate the occurrence of anxiety in animals, the OF test was performed. The SNI animals spent less time in the center of the arena than the Sham animals, suggesting a strong state of anxiety-like behavior. SNI animals treated with RA 5 mg/kg increased their time spent in the center of the arena, indicating an anxiolytic-like effect of RA (Figure 4D).

This was confirmed with the LDB test. The treatment with RA reduced the anxiety state of the animals by increasing their time spent in the light compartment (Figure 4E). No effect was observed on the number of transitions from one part to the other (Figure 4F).

In addition to the assessment of anxiety-like symptoms, we also assessed the occurrence of symptoms associated with depression by performing the SST. In a relaxed state, the mouse will tend to take more care of its appearance and will clean itself more frequently (Figure 4G). SNI animals spent less time on cleaning compared to non-operated mice (Sham). Treatment with RA was able to attenuate this depressant-like tendency in operated animals by slightly increasing the time spent by the mice cleaning themselves. This antidepressant-like effect was more evident with the TST. During the 6 min of the test, in the first 2 min, the basal reaction of the animal towards aversive conditions, and the next 4 min, in which a behavioral despair is established making it possible to evaluate the antidepressant-like effect of a drug, were initially measured (Figure 4H). In the final 4 min of the test, the SNI animals remained immobile for a longer time, compared to the Sham, while treatment with RA restored the values to the control level (Figure 4H).

### 3.5. RA Reduced the Expression of β-Galactosidase in the Spinal Cord and Hippocampus of SNI Mice

**Figure 5:**
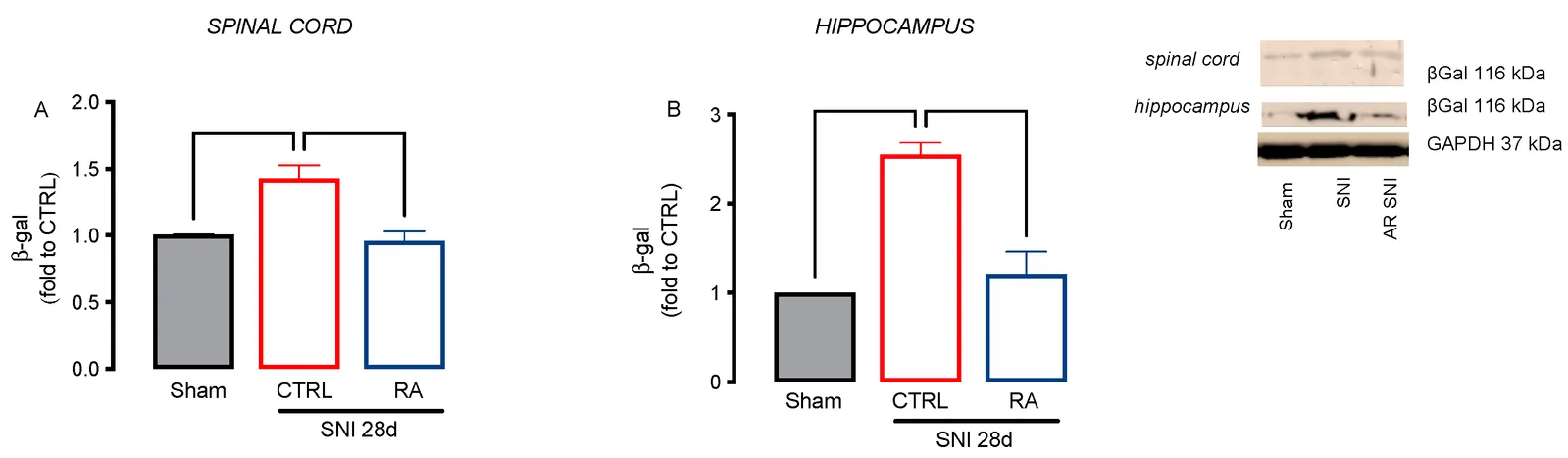
Effect of RA on ß-gal expression. β-galactosidase (ß-gal) protein expression in spinal cord tissue (**A**) and hippocampus (**B**) in un-operated mice (Sham), mice with neuropathy (SNI), and mice with neuropathy treated with rosmarinic acid (RA) 5 mg/kg after 28 days from surgery. Representative blots are reported, loading sample individually.

After showing the relationship of neuroinflammation and cellular senescence in vitro, we investigated their correlation with NP by evaluating the presence of these processes in biological tissues from SNI animal models. The study involved samples from the spinal cord and hippocampus, which are mainly involved in pain and associated comorbidities. Concomitant with clinical symptoms, an elevated level of β-gal in the spinal cord (Figure 5A) and hippocampus (Figure 5B) of SNI mice was observed, compared to sham-operated controls, suggesting the development of cellular senescence in these tissues (Figure 5A). The administration of RA reduced the expression of this marker in both tissues.

## 4. Discussion

The incidence of NP worldwide is around 7–8%, with greater frequency in women than men (8% versus 5%, respectively) and in patients over 50 years of age (8.9% versus 5.6% for those under) [29,30]. This is accompanied by several comorbidities such as anxiety, depression, memory impairment, and sleep disturbances. These factors have an important negative influence on the neuropathic condition itself and consequently on the patient’s quality of life, requiring a therapeutic approach that acts on several fronts [2,31]. Today, the first line of treatment for NP is represented by TCAs, antiepileptic drugs such as gabapentin or pregabalin, and serotonin-norepinephrine reuptake inhibitors (SNRIs) [31]. However, these treatments do not address the underlying causes of neuropathy but are symptomatic. Furthermore, the side effects brought by these drugs, such as sedation associated with TCAs, should not be forgotten and can further compromise the quality of the patient’s life [32]. Consequently, in recent years studies have focused on finding parallel therapies that could complement or in some cases even replace the classical ones. Natural products fit into this context, especially considering their heterogeneity of the mechanisms of action [33]. Formulations based on *Melissa officinalis* L., known to be therapeutically effective as a sedative or calming agent in mood disorders such as anxiety, depression, and insomnia, have been widely used for centuries in various pathological contexts. These conditions are also the main comorbidities of the NP (Kennedy et al., 2004). RA is a natural antioxidant and anti-inflammatory polyphenol derived from many plants including *M. officinalis*. Polyphenols are characterized by numerous beneficial effects on the body, and RA, with its numerous properties, makes no exception, especially as a strong anti-inflammatory [34,35,36,37]. Indeed, RA was reported to exert anti-inflammatory effects against neuroinflammation in microglia cells [38]. Neuroinflammation is one of the processes underlying the neuropathic condition. Following peripheral neuronal injury, a major inflammatory response develops, linked to the activation of microglia, which are thought to be one of the main contributors to the hypersensitivity characteristic of neuropathy [12,39,40] The chronic microgliosis that characterizes NP then leads to a loss of activity of these cells, which can no longer recover their physiological activity, leading the cell towards senescence. Senescent microglia have recently been linked with the onset of numerous neuronal pathologies, including chronic pain. The aim of this work, hence, was to test whether RA could alleviate the NP and NP-associated symptoms in the SNI neuropathy and to test whether these effects can be related to a reduction of microglial senescence. In our work, we optimized the concentration of RA required for the best activity in reducing inflammation in LPS-stimulated (24 h) microglial cells, resulting in a pro-inflammatory microglial phenotype. RA was found to be active in counteracting neuroinflammation even at very low micromolar concentrations. These results are consistent with Wei and coworkers [38], who showed the ability of RA to suppress pro-inflammatory microglial activation and to promote microglial polarization to the anti-inflammatory phenotype in LPS-stimulated BV2 cells. Previously, it has been reported that RA suppressed TLR4 and CD14, reducing inflammasome activation [41]. Given the activity on neuroinflammation, we then moved on to test the concentration of RA needed for reducing inflammation in the microglial senescence model. An in vitro model of senescence based on BV-2 cell cultures, in which cells are stimulated every 48 h for a duration of 4 h with LPS, up to six total stimulations was already reported in the literature [16]. By modifying the timing and concentration of LPS, we optimized the microglial senescence model and obtained more robust effects. In our model, BV-2 cells were stimulated with LPS 500 ng/mL every 72 h for 4 h/day, over a period of 10 days. It was possible to confirm an increase in the senescent phenotype by considering several specific markers of senescent microglia, such as increased levels of the enzyme β-gal, increased release of SASP factors, decreased cell viability and the presence of nuclear foci of senescence. Applying RA to the model a reduction in each parameter was observed, indicating a reduction in the cellular ageing process. Protective effects of RA against oxidative cellular senescence have been already observed on fibroblasts [42] and neurons [43], but this is the first work in which the anti-senolytic activity of RA against microglial cells was observed. Medicinal plant extracts with high amounts of RA have also been seen to have anti-ageing properties in the clinical practice. A clinical study in the literature has shown that chronic administration of a lemon balm extract in a daily dose of 500 mg improves the cognitive abilities of patients suffering from Alzheimer’s disease with mild associated dementia, with a reduction in the cellular senescence process. Intriguingly, the used extract had a high RA content [44]. Once we had assessed the effect of the RA on neuroinflammation and microglial senescence using the in vitro model, we moved on to test it in an in vivo model of peripheral neuropathy, the SNI. In this model, RA was able to reduce not only the hyperalgesia produced by the SNI, but also the anxiety and depression associated with it. Indeed, it is very common for patients with neuropathy to have comorbidities that debilitate their normal quality of life. A correlation between relief of SNI symptoms and attenuation of microglia senescence was highlighted by the reduction of senescence markers at both hippocampal and spinal level, the main players involved in pain/anxiety/depression mechanisms, following RA oral treatment. Present data indicate that, despite RA’s low ability to cross the blood-brain barrier, the oral dose used of 5 mg/kg repeatedly administered was sufficient to have a central effect [45]. As previously reported, after a single oral administration, RA could be quickly absorbed into the blood and eliminated slowly [46]. In addition, our data are consistent with those of Areti and colleagues, who showed that RA 25 mg/kg p.o. reduced spinal glia activation in oxaliplatin-induced peripheral neuropathy, leading to a reduction of mechanical allodynia and cold hyperalgesia [47]. Moreover, RA 10 and 30 mg/kg was able to increase the paw withdrawal threshold of diabetic rats in a model of streptozotocin (STZ)-induced neuropathy [48] and, in a model of chronic constriction injury (CCI), orally administered RA was able to reduce heat hyperalgesia up to fourteen days after the operation [49].

Finding an orally administered therapy that can reduce both hyperalgesia and other symptoms associated with neuropathy is certainly an important objective in clinical practice. RA has been shown to lower cognitive disorders in several animal models [49,50], further supporting the efficacy of RA in relieving both NP and associated symptoms reported in the present study. Indeed, for the first time we have seen that RA is able to control both hyperalgesia and behavioral disturbances, which is certainly a pharmacological advantage. The final stage of research was to assess the development of cellular senescence in biological tissues from SNI mice and to correlate RA efficacy in attenuating SNI phenotype with microglia senescence. It is therefore hypothesized that a cellular ageing process develops in these tissues during neuropathy. However, it is not known whether this occurs, and which cells are affected, so the aim for the future is to carry out further studies.

## 5. Conclusions

The use of natural substances as a nutraceutical intervention for the prevention or treatment of numerous diseases affecting the central nervous system leads to the study and research of new bioactive molecules. In this paper, we investigated the role of RA, a typical bioactive molecule found in many medicinal plants used in traditional medicine as potent anti-inflammatory agents, for its possible senolytic activity. This last aspect turns out to be very interesting, as cellular aging appears to be central in the onset of many diseases. In this work, we specifically wanted to investigate microglial senescence in the modulation of neuroinflammation related to neuropathic pain. Indeed, the loss of activity of these cells impairs the normal function of the nervous system. In our work, RA could reduce inflammation and microglial senescence in vitro, and these activities may be related to its ability to reduce symptoms associated with peripheral neuropathy in the murine SNI model. Although further studies are required to investigate the role of RA in pain/behavioral disorders/senescence in more detail, this study represents a starting point for investigating the role of microglial senescence as a possible pharmacological target in controlling symptoms related to the later stages of peripheral neuropathy.

## Data Availability

Not applicable.
