# Correction: Borgonetti, V.; Galeotti, N. Rosmarinic Acid Reduces Microglia Senescence: A Novel Therapeutic Approach for the Management of Neuropathic Pain Symptoms. *Biomedicines* 2022, *10*, 1468

**DOI:** 10.3390/biomedicines14092023

**Published:** 2026-09-09

**Authors:** Vittoria Borgonetti, Nicoletta Galeotti

**Affiliations:** Department of Neuroscience, Psychology, Drug Research and Child Health (NEUROFARBA), Section of Pharmacology, University of Florence, 50139 Florence, Italy; vittoria.borgonetti@unifi.it

## Text Correction

In the original publication [1], the catalogue number of anti-ß-galactosidase was cited as “Cat# sc-65670, RRID:AB_831022IBA1”, instead of the correct product information, “Cat# sc-377257”.

A correction has been made to the respective sentence in Section 2.11 “Western Blotting”, Paragraph 1:

“Membranes were blocked in PBST (PBS with 0.1% Tween) containing 5% non-fat dry milk for 90 min and incubated overnight at 4 °C with primary antibodies: anti-ß-galactosidase (Santa Cruz Biotechnology, Dallas, TX, USA, Cat# sc-377257), anti-IκBα (Santa Cruz Biotechnology, Dallas, TX, USA, Cat# sc-1643, RRID:AB_627772), and anti-IL-1ß (Santa Cruz Biotechnology, Dallas, TX, USA, Cat# sc-52012, RRID:AB_629741)”.

The authors state that the scientific conclusions are unaffected. This correction was approved by the Academic Editor. The original publication has also been updated.
